# Status Epilepticus in an Internal Medicine Ward: Different Patients Therefore Distinct Approaches

**DOI:** 10.7759/cureus.34259

**Published:** 2023-01-27

**Authors:** Miguel Trindade, Mafalda Teixeira, João Serôdio, Catarina Favas, José Delgado Alves

**Affiliations:** 1 Department of Internal Medicine IV, Hospital Professor Doutor Fernando Fonseca, Amadora, PRT; 2 Department of Internal Medicine IV, Systemic Immune-Mediated Diseases Unit (UDIMS), Hospital Professor Doutor Fernando Fonseca, Amadora, PRT; 3 Department of Internal Medicine, Nova Medical School, Lisbon, PRT

**Keywords:** status epilepticus, epilepsy research, general internal medicine, elderly population, non-convulsive status epilepticus

## Abstract

Background

Status epilepticus (SE) is a medical condition that bestows substantial morbidity and mortality. Literature is scarce regarding SE in elderly patients, particularly in the context of internal medicine wards.

Aim

To characterize SE patients admitted to an internal medicine ward, identify potential outcome predictors and differences between young and elderly, as well as convulsive (CSE) and non-convulsive SE (NCSE) patients.

Methods

We enrolled 135 consecutive patients in an observational, retrospective cohort study. We established elderly patients as more than 64 years old and defined worse prognosis as a modified Rankin Scale (mRS)>4.

Results

The SE population was 73% elderly, and 75% presented with NCSE, mainly metabolic, idiopathic, or vascular SE. The intra-hospital mortality was 51%, and 62% had an mRS>4 at discharge. NCSE and electroencephalogram (EEG) with paroxysmal activity at discharge were predictive of a worse prognosis. Elderly patients had increased disability at admission, most had NCSE (81%), and the SE etiology differed with more idiopathic and vascular causes. In the elderly, mortality was increased, as was the number of patients with mRS>4 at discharge. NCSE patients had the more neurodegenerative disease (30%) and presented predominantly with vascular and anoxic causes. Morbidity and mortality were also increased in the NCSE group. There was no difference in the antiepileptic drugs used or in the percentage of patients achieving an EEG with no paroxysmal activity between the subpopulations.

Conclusion

SE in elderly patients should be addressed distinctly. Current approaches based on the strategies used for standard CSE have shown little or no efficacy overall.

## Introduction

Status epilepticus (SE) was defined in 2015 by the International League Against Epilepsy (ILAE) as a condition resulting either from the failure of the mechanisms responsible for the seizure to terminate or from the initiation of mechanisms that lead to abnormally prolonged seizures [1]. SE is a condition that can have long-term consequences, including neuronal death, neuronal injury, and modification of the neuronal networks, depending on the type and duration of seizures [1].

SE incidence is increasing in population-based studies worldwide due to the broader availability of electroencephalograms (EEG), more precise diagnostic criteria, and an aging population [2]. Furthermore, SE, particularly non-convulsive SE (NCSE), is increasingly recognized among patients older than 60 years old (elderly associated SE - (EASE)) [3]. NCSE is also frequently superimposed on other acute medical illnesses in the elderly [4,5]. However, the pathophysiology is poorly understood, and the consequent management of SE among the elderly is far from optimal. It is unclear whether this clinical entity has any common mechanisms with the SE present in young patients with epilepsy, and it is also unclear if traditional antiepileptic drugs are effective among these patients.

Moreover, the correct management of SE often requires complex treatments and admission to the intensive care unit (ICU), which may be questionable in elderly, comorbid frail patients [6]. Consequently, EASE has substantial morbidity and mortality [4,5]. Nevertheless, literature is scarce regarding SE in elderly patients, particularly in the context of internal medicine wards.

## Materials and methods

We conducted an observational, retrospective single-center cohort study to characterize the population of patients with SE admitted to an internal medicine ward and identify potential outcome predictors. We also aimed to identify clinical differences between SE in young and elderly patients and between convulsive (CSE) and NCSE.

We enrolled 135 consecutive patients with SE admitted to an internal medicine ward in our department between 2017 and 2020. We classified SE from the clinical point of view as CSE or NCSE. CSE was defined as a seizure that lasted longer than 5 minutes or had more than one seizure within 5 minutes without returning to a normal level of consciousness between episodes. We defined NCSE as an altered mental state with minimal rhythmic motor activity or a stuporous/comatose state and an EEG with paroxysmal activity consistent with NCSE according to the Salzburg criteria [7].

We collected demographic and clinical data, neuro-imaging studies, and EEG data. The patient's functional status was defined by the modified Rankin Scale (mRS) and was assessed prior to hospital admission and at discharge. We established elderly patients as more than 64 years old. We defined a bad outcome as total disability or death at discharge corresponding to mRS>4.

Descriptive results for continuous variables were expressed as mean and standard deviation or as median and interquartile range, depending on the normality of their distribution. Variables were tested for their association with prognosis using Pearson's chi-squared test for categorical data and the Mann-Whitney U-test for numerical data. A multiple stepwise logistic regression model was established with any covariate with the univariate significance of a P-value less than 0.05 eligible for inclusion in the model.

## Results

A total of 135 SE patients were included. Their baseline characteristics are displayed in Table 1. They were predominantly female (58%) with a median age of 72 (64-81) years old, and 73% were elderly. The median mRS at admission was 3 (0-5). One hundred and one patients (75%) presented with NCSE and 34 (25%) with CSE.

**Table 1:** Demographic and clinical variables collected in the status epilepticus population. A comparison between elderly and young patients and non-convulsive and convulsive status epilepticus is also presented. *Number of neuroimaging studies available = 124; *^2^ number of electroencephalogram available = 115 SE - status epilepticus; NCSE - non-convulsive status epilepticus; CSE - convulsive status epilepticus; CI - confidence interval; mRS - modified Rankin Scale; ICU - intensive care unit; CNS - central nervous system; EEG - electroencephalogram

Variables	SE population	Young	Elderly	p	NCSE	CSE	p
n (%)	135 (100%)	36 (27%)	99 (73%)	-	101 (75%)	34 (25%)	-
Female, n (%)	78 (58%)	11 (31%)	67 (68%)	<0.001	64 (63%)	14 (41%)	0.020
Age at diagnosis (years), median (95% CI)	72 (64-81)	52 (43-60)	77 (70-84)	-	74 (65-82)	66 (51-77)	0.002
mRS at admission, median (95% CI)	3 (0-5)	1 (0-3)	3 (1-5)	<0.001	3 (1-5)	1 (0-3)	0.006
Non convulsive status epilepticus, n (%)	101 (75%)	21 (58%)	80 (81%)	0.008	-	-	-
Time to discharge (days), median (95% CI)	17 (7-29)	17 (5-41)	17 (7-28)	0.729	17 (7-35)	13 (4-22)	0.199
ICU admission, n (%)	44 (33%)	19 (53%)	25 (25%)	0.003	24 (24%)	20 (59%)	<0.001
Mechanical ventilation, n (%)	35 (26%)	16 (44%)	19 (19%)	0.003	20 (20%)	15 (44%)	0.005
Cerebrovascular disease, n (%)	42 (31%)	7 (19%)	35 (35%)	0.077	36 (36%)	6 (18%)	0.050
Neurodegenerative disease, n (%)	34 (25%)	6 (17%)	28 (28%)	0.169	30 (30%)	4 (12%)	0.037
Epilepsy (previous diagnosis), n (%)	34 (25%)	13 (36%)	21 (21%)	0.078	26 (26%)	8 (24%)	0.797
CNS Infection (previous diagnosis), n (%)	9 (6.7%)	5 (14%)	4 (4%)	0.042	4 (4%)	5 (15%)	0.030
CNS cancer, n (%)	5 (3.7%)	2 (5.6%)	3 (3%)	0.492	5 (5%)	0 (0%)	0.186
CNS metastasis, n (%)	9 (6.7%)	2 (5.6%)	7 (7.1%)	0.755	4 (4%)	5 (15%)	0.030
CNS trauma (previous diagnosis), n (%)	5 (3.7%)	3 (8.3%)	2 (2%)	0.086	2 (2%)	3 (8.8%)	0.068
Chronic Ischemic Leukoencephalopathy, n (%)*	84 (68%)	12 (39%)	72 (77%)	<0.001	70 (75%)	14 (47%)	0.005
Cerebral atrophy, n (%)*	34 (27%)	3 (9.7%)	31 (33%)	0.011	25 (26%)	9 (29%)	0.791
≥ 1 Cardiovascular Risk Factor, n (%)	99 (73%)	16 (44%)	83 (84%	<0.001	78 (77%)	21 (62%)	0.078
High blood pressure, n (%)	85 (63%)	10 (28%)	75 (76%)	<0.001	65 (64%)	20 (59%)	0.563
Dyslipidaemia, n (%)	48 (36%)	5 (14%)	43 (43%)	0.002	37 (36%)	11 (32%)	0.652
Diabetes mellitus, n (%)	39 (29%)	2 (5.6%)	37 (37%)	<0.001	33 (33%)	6 (18%)	0.095
Atrial fibrillation, n (%)	7 (6.7%)	7 (7%)	0 (0%)	0.101	6 (6%)	1 (3%)	0.495
Coronary artery disease, n (%)	12 (8.9%)	2 (5.6%)	10 (10%)	0.412	10 (9.9%)	2 (5.9%)	0.476
Obesity, n (%)	18 (13%)	3 (8.3%)	15 (15%)	0.303	17 (17%)	1 (2.9%)	0.039
Tobacco smoking users, n (%)	10 (7.4%)	3 (8.3%)	7 (7.1%)	0.804	8 (7.9%)	2 (5.9%)	0.695
Excessive alcohol consumption (>40g), n (%)	18 (13%)	8 (22%)	10 (10%)	0.067	11 (11%)	7 (21%)	0.150
Cancer (non-CNS), n (%)	8 (5.9%)	0 (0%)	8 (8.1%)	0.079	6 (5.9%)	2 (5.9%)	0.990
EEG available, n (%)	115 (85%)	30 (83%)	85 (86%)	0.715	87 (86%)	28 (82%)	0.591
EEG with paroxysmal activity at discharge, n (%)*^2^	70 (52%)	17 (59%)	53 (62%)	0.721	55 (64%)	15 (54%)	0.327
Levetiracetam, n (%)	120 (89%)	31 (86%)	89 (90%)	0.536	92 (91%)	28 (82%)	0.161
Valproic acid, n (%)	81 (60%)	22 (61%)	59 (60%)	0.874	56 (55%)	25 (74%)	0.063
Phenytoin, n (%)	17 (13%)	6 (17%)	11 (11%)	0.390	7 (6.9%)	10 (29%)	0.001
Lacosamide, n (%)	24 (18%)	6 (17%)	18 (18%)	0.839	17 (17%)	7 (21%)	0.620
Clobazam, n (%)	27 (20%	7 (19%)	20 (20%)	0.922	24 (24%)	3 (8.8%)	0.060
Antiepileptic drugs used, median (95% CI)	2 (1-3)	3 (1-4)	2 (1-3)	0.160	2 (1-3)	3 (2-4)	0.058
Levetiracetam as the first drug used, n (%)	68 (50%)	18 (50%)	50 (51%)	0.756	54 (54%)	14 (41%)	0.074
Valproic acid as the first drug used, n (%)	33 (24%)	8 (22%)	25 (25%)	0.756	22 (22%)	11 (32%)	0.074
≥ 1 Nosocomial Infection, n (%)	62 (46%)	12 (33%)	50 (51%)	0.077	47 (47%)	15 (44%)	0.807
Death, n (%)	69 (51%)	11 (31%)	58 (59%)	0.004	57 (56%)	12 (35%)	0.033
mRS at discharge, median (95% CI)	5 (3-6)	3,5 (1-6)	6 (4-6)	<0.001	6 (4-6)	3,5 (1-6)	0.001
mRS > 4 at discharge, n (%)	83 (62%)	14 (39%)	69 (70%)	0.001	71 (70%)	12 (35%)	<0.001

The median time to discharge was 17 (7-29) days, 33% required ICU admission, and 26% were placed on mechanical ventilation. Before admission, at least one cardiovascular risk factor was present in 76% of patients, and a neurodegenerative disease was present in 25% of patients. Imaging exams revealed chronic ischemic leukoencephalopathy in 68% and cerebral atrophy in 27% of the patients (n=125; 93%). One-third of the patients achieved an EEG without paroxysmal activity at discharge. The median number of antiepileptic drugs used was two (1-3), mostly levetiracetam (89%) and valproic acid (60%). Nosocomial infection was an in-hospital complication in 46% of patients. The mortality in the overall SE population was 51%, and 62% had an mRS>4 at discharge.

The cause of SE was distributed as metabolic (25%), idiopathic (24%), vascular (20%), structural (13%), anoxia (7%), central nervous system (CNS) infection (7%), and others (5%) as displayed in Table 2.

**Table 2:** Status epilepticus etiology in the study population. SE - status epilepticus; NCSE - non-convulsive status epilepticus; CSE - convulsive status epilepticus

Status Epilepticus Etiology	SE population	Young	Elderly	p	NCSE	CSE	p
Metabolic	34 (25%)	9 (25%)	25 (25%)	0.004	24 (24%)	10 (29%)	0.022
Idiopathic	32 (24%)	6 (16%)	26 (26%)		26 (26%)	6 (18%)	
Vascular	27 (20%)	3 (8.3%)	24 (24%)		24 (24%)	3 (8.8%)	
Structural	17 (13%)	6 (17%)	11 (11%)		10 (9.9%)	7 (20%)	
Anoxia	9 (6.7%)	4 (11%)	5 (5.1%)		9 (8.9%)	0 (0%)	
Central nervous system infection	9 (6.7%)	2 (5.6%)	7 (7.1%)		4 (4.0%)	5 (15%)	
Others	7 (5.2%)	6 (17%)	1 (1%)		4 (4.0%)	3 (8.8%)	

Elderly patients were primarily female (68% vs. 31%, p>0.001) and had increased disability at admission (median mRS 3 (1-5) vs. 1 (0-3), p<0.001). Most had NCSE (81% vs. 58%, p=0.008), and the SE etiology was significantly different (p=0.04), with more idiopathic and vascular-associated causes and fewer anoxia-associated causes (Table 2). They also had more cardiovascular risk factors (84% vs. 44%, p>0.001), chronic ischemic leukoencephalopathy (77% vs. 39%, p<0.001), and cerebral atrophy (33% vs. 9.7%, p=0.011). The presence of previous CNS infection was also inferior in the elderly population (4% vs. 14%, p=0.042).

Elderly patients had fewer admissions to ICU (25% vs. 53%, p=0.003) and indications for mechanical ventilation (19% vs. 44%, p= 0.003). There was no difference in the drug treatment for SE, with the same antiepileptic therapy being also used in younger patients. Likewise, EEG without paroxysmal activity at discharge was similarly achieved in the same proportion of younger and elderly patients (38% vs. 41%, p=0.721). Mortality was increased in the elderly population (59% vs. 31%, p=0.004), as was mRS>4 at discharge (70% vs. 39%, p=0.001).

NCSE patients were older (74 (65-82) vs. 66 (51-77) years old, p=0.002) and presented more frequently previous incapacity (median mRS 3 (1-5) vs. 1 (0-3), p=0.006). They also had more neurodegenerative disease (30% vs. 12%, p=0.03), chronic ischemic leukoencephalopathy (74% vs. 47%, p<0.01), obesity (17% vs. 2.9%, p=0.039), and less previous history of CNS infection (4% vs. 15%, p=0.030) or CNS metastasis (4% vs. 15%, p=0.030). NCSE causes were significantly different, with a predominance of vascular (24% vs. 8.8%) and anoxic (8.9% vs. 0%) causes. SE caused by structural (20% vs. 9.9%) or CNS infection (15% vs. 4%) was primarily convulsive (Table 2).

Both ICU admission (59% vs. 24%, p<0.001) and mechanical ventilation (44% vs. 20%, p=0.005) were more frequent in CSE, as was the use of phenytoin (29% vs. 6.9%, p=0.001). There was no difference in the number of antiepileptic drugs used or in the percentage of patients achieving an EEG with no paroxysmal activity. Morbidity and mortality were increased in the NCSE group (mRS>4 in 70% vs. 35%, p<0.001 and in-hospital death 56% vs. 35%, p=0.033).

In a univariate analysis, age, mRS at admission, NCSE, neurodegenerative disease, previous CNS infection, tobacco user, excessive alcohol consumption, and an EEG with paroxysmal activity at discharge were associated with a bad outcome (mRS>4) as displayed in Table 3. On multivariate analysis, only NCSE (OR 1.217; 95% CI 1.170-9.738; p=0.024) and EEG with paroxysmal activity at discharge (OR 2.583; 95% CI 1.039-6.424; p=0.041) remained significantly predictive of a worse prognosis. The mRS at admission was not considered for multivariate analysis due to its direct dependence on the autonomy and mortality composite defined as a worse outcome (mRS at discharge).

**Table 3:** Univariate and multivariate analysis for worse prognosis defined by a modified Rankin Scale ≥ 4. SE - status epilepticus; NCSE - non-convulsive status epilepticus; CSE - convulsive status epilepticus; OR - odds ratio; CI - confidence interval; mRS - modified Rankin Scale; CNS - central nervous system; EEG - electroencephalogram

Variable	Univariate analysis (mRS >4)				Multivariate analysis (mRS >4)			
	OR	95% CI		p	OR	95% CI		p
Female sex	0.770	0.382	1.551	0.465				
Age at diagnosis	1.046	1.019	1.073	0.001	0.025	0.994	1.058	0.116
mRS at admission	1.456	1.199	1.767	<0.001				
Non convulsive status epilepticus	4.339	1.906	9.878	<0.001	1.271	1.170	9.738	0.024
Cerebrovascular disease	0.621	0.287	1.345	0.227				
Neurodegenerative disease	3.903	1.487	10.242	0.006	1.153	0.098	1.017	0.053
Epilepsy (previous diagnosis)	0.832	0.371	1.867	0.655				
CNS Infection (previous diagnosis)	6.300	1.255	31.618	0.025	0.485	0.238	11.093	0.621
CNS cancer	0.387	0.042	3.563	0.402				
CNS metastasis	1.300	0.333	5.081	0.706				
CNS trauma (previous diagnosis)	1.067	0.172	6.661	0.945				
Chronic Ischemic Leukoencephalopathy	0.646	0.302	1.386	0.262				
Cerebral atrophy	0.610	0.266	1.397	0.242				
≥ 1 Cardiovascular Risk Factor	0.835	0.384	1.818	0.651				
Tobacco smoking users	4.148	1.022	16.837	0.047	1.059	0.443	18.770	0.268
Excessive alcohol consumption (>40g)	5.200	1.730	15.633	0.003	1.320	0.063	1.132	0.073
Cancer (non-CNS)	0.513	0.100	2.645	0.425				
EEG with paroxysmal activity at discharge	2.871	1.314	6.271	0.008	2.583	1.039	6.424	0.041
≥ 1 Nosocomial Infection	1.151	0.574	2.307	0.691				

## Discussion

In this study, we identified 135 patients with SE, primarily elderly women with manifestations of cardiovascular disease and at least moderate disability (mRS=3). NCSE was predominant, and the metabolic and vascular etiologies accounted for half of the study population. This population differs entirely from the one described in the sustaining SE management recommendations presented in published guidelines [8-10]. The patients enrolled in the largest randomized controlled trial (RCT) regarding anticonvulsant medications for SE were mainly men with a median age of 33±25 years old, a combined vascular and metabolic etiology of 21.5%, and in whom metabolic, liver, or kidney disease were exclusion criteria together with NCSE [11]. The recognition that our population is sub-represented in the literature propelled us to characterize it to increase our understanding of this pathology and help achieve better outcomes.

The morbidity and mortality in the study population were meaningful, with 41% developing a nosocomial infection and 61% severely disabled or dead before discharge. Roughly 75% required at least two anticonvulsive drugs, with levetiracetam and valproic acid being the most used. NCSE was independently associated with a worse prognosis. Due to the absence of RCT regarding NCSE drug therapy, our utilization of levetiracetam, valproic acid, and others is guided mainly by recommendations for CSE [4]. The persistence of paroxysmal activity in the EEG was also associated with a worse prognosis in the multivariate analysis, establishing a therapeutic target to achieve in this population.

The main features of our population were older age and the preponderance of NCSE. This observation concurred with previous works regarding SE in internal medicine wards [12,13]. As such, we opted further to analyze the elderly patients and NCSE in our study population.

As expected, elderly patients had more frailty and disability at admission and more cardiovascular disease, including chronic ischemic leukoencephalopathy and cerebral atrophy. They were primarily women with NCSE due to metabolic, idiopathic, and vascular etiologies. Even though they were almost exclusively treated in internal medicine wards without admission to ICU, there was no difference in the drug treatment, and the number of patients with an EEG without paroxysmal activity at discharge was similar between the younger and elderly. Despite that, disability at discharge and mortality were superior in the last group.

Compared to CSE, NCSE patients are older, frailer, and more disabled, probably due to increased neurodegenerative disease and vascular CNS disease. Vascular and metabolic causes were predominant, and CNS infection and structural diseases like metastasis were not characteristic of these patients, contrary to what happens in CSE. Drug treatment was roughly the same except for increased phenytoin use in CSE. Achieving EEG with no paroxysmal activity was not affected by treatment as it was equal between CSE and NCSE, but morbi-mortality was superior for NCSE patients.

The diagnosis of NCSE has been increasing, probably due to an increase in awareness and the complexity of the patients admitted to internal medicine wards. In fact, it has been predicted a steady increment in the diagnosis of NCSE in the years to come [2]. Our results suggest that NCSE and CSE have significant clinical and etiologic differences that should translate into different approaches. At the same time, elderly patients with SE differ from young ones, and a parallel has been established between NCSE and increasing age - EASE.

Evidence suggests that systemic dysfunctions such as acute inflammation or non-CNS infections may trigger neurologic dysfunction in susceptible patients, making NCSE, particularly in EASE patients, a neurologic manifestation of a broader systemic disease. In mouse models of Alzheimer's disease, β-amyloid was demonstrated to favor epileptiform activity, even at early stages of the disease process and in the absence of overt neuronal loss [14]. However, few studies have focused on the development of epilepsy in aged animal models [15,16]. Compared to younger, aged animals were more susceptible to induced seizures, typically more severe and leading to a greater degree of hippocampal degeneration [15]. Moreover, aged mice exhibited a lower threshold for seizures when different drugs were used to induce them (kainic acid, pilocarpine, nicotine) and were more sensitive to the GABAergic (gamma-aminobutyric acid) anticonvulsant action of benzodiazepines like oxazepam [17,18]. Investigation in specific mice models demonstrated a general reduction of transcriptional responses for several proteins following induced seizures in the aged brain. It may be directly related to the limited plasticity, and functional recovery often observed in injured aged brain tissue [15].

Regarding inflammation and epilepsy, proinflammatory cytokines like interleukin-1β and interleukin-6 induced by bacterial lipopolysaccharides (LPS) were linked to the control of thalamocortical excitability by exerting substantial effects on physiological synchronization such as sleep and pathological synchronization such as the one present in epileptic discharges [19]. Furthermore, LPS injection induced absence seizures by induction of prostaglandin synthesis and prostaglandin action, suggesting some common targets in epilepsy and lipopolysaccharide-induced inflammation, such as inflammatory cytokines and excitatory neurotransmitters [19,20].

Although some information suggests that aged animals may be more susceptible to seizures, it is essential to note that investigational antiepileptic drugs have not been tested on them. Most anticonvulsant drugs target ion channels and neurotransmitter receptors linked to neuronal excitability but do not possess disease-modifying properties or actual anti-epileptogenic activity. More recent trends are focusing on the identification of primary cell signaling pathways that trigger the downstream mechanism of epilepsy, such as the mammalian target of rapamycin (mTOR) pathway, which is increasingly recognized to be involved in a diverse group of human diseases such as cancer, cardiovascular diseases, diabetes, obesity, and neurological disorders [21]. Chronic treatment with rapamycin/sirolimus, a macrolide with anti-tumor and immunosuppressive activity on the mTOR pathway, was able to block LPS-inducted absence seizures, further suggesting an anti-epileptogenic inflammatory-like protective action [21].

Therapeutic goals in a significant number of EASE patients should probably be directed towards the metabolic or inflammatory overall disturbance and not be exclusively based on anticonvulsant medication. Nevertheless, on the grounds of current knowledge, achieving an EEG without paroxysmal activity is still considered a therapeutic goal that should be persecuted as soon as the diagnosis is made.

To our knowledge, ours is the largest series of SE in internal medicine wards, with the most comprehensive data regarding demographic, functional status, clinical data, EEG, and neuroimaging studies. A potential limitation of our study is the absence of a control group and the retrospective methodology.

Following the recognition that EASE might have fundamental differences from CSE in younger patients, the treatment of established status epilepticus in the elderly (ToSEE) trial, the first prospective randomized and multicenter trial in SE in the elderly population, was started in 2020 and is currently recruiting [22]. It aims to directly evaluate levetiracetam against valproic acid for acute seizure termination (60 minutes) in CSE and NCSE patients more than 64 years old. It will provide the first evidence-based approach to the management of our SE population, although it is still limited to anticonvulsant drugs. Therefore, more evidence is needed regarding therapies that act on the signaling pathways responsible for the lower threshold of seizure development in these patients. This step seems critical in blocking paroxysmal activity due to acute medical causes and preventing the disability and mortality associated with EASE.

## Conclusions

The diagnosis of NCSE has been increasing in patients admitted to internal medicine wards. These patients are primarily elderly, frail, and present with increasing disabilities. SE in these patients is predominantly associated with inflammation and metabolic disturbances, and anticonvulsant medication is usually ineffective. Approaching these patients like a young patient with a structural or genetic neurologic abnormality distances us from the primary dysfunction. Therapeutic goals in these populations should be directed toward metabolic or overall inflammatory disturbance.

We believe SE in elderly patients fundamentally differs from standard CSE and should be addressed distinctly. Current approaches based on the strategies used for standard CSE have shown little or no efficacy overall. Evidence from SE in aged animal models, the influence of the mTOR pathway, and the anti-epileptogenic inflammatory-like protective action of sirolimus should be followed up. We ought to change from targeting neurotransmitter receptors linked to neuronal excitability to disease-modifying properties and/or actual anti-epileptogenic activity.
